# Supplementary material for: Downsizing a heavyweight: factors and methods that revise weight estimates of the giant fossil whale Perucetus colossus
Source: PeerJ. 2024 Feb 29;12:e16978. doi: 10.7717/peerj.16978 (PMC10909350; doi:10.7717/peerj.16978)
Supplement: Supplemental Information 1 [file peerj-12-16978-s001.zip › Supplementary Figure 1.pdf]

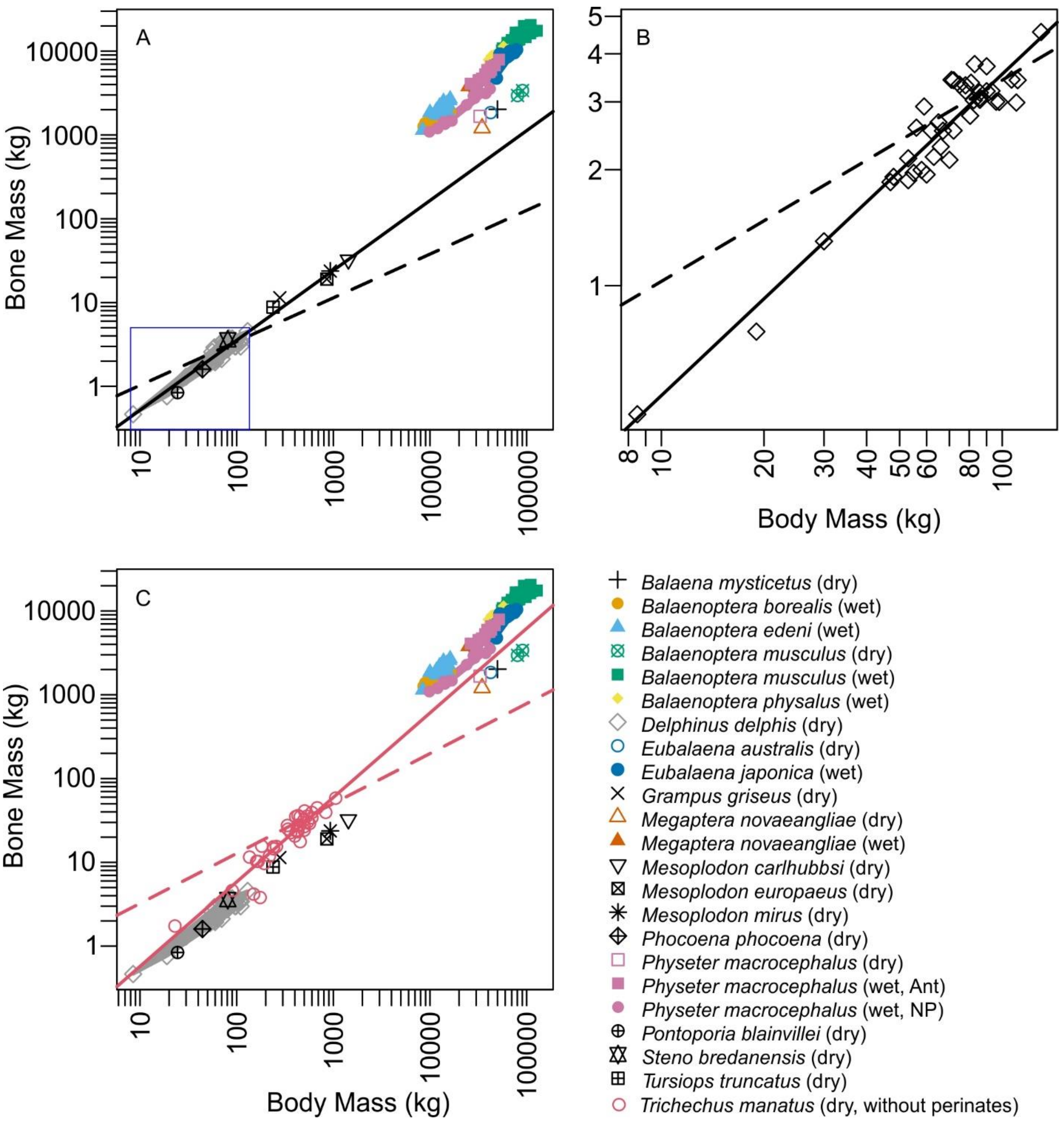

Supplementary Figure 1. Bias from using adult data only, based on Fig. 4. **A**, Regression lines for all data points (solid) versus adults only (dashed) for *Delphinus delphis*. **B**, same as A but zoomed in to the blue rectangle to show that adults have more scatters around the regression line than juveniles. **C,** Regression lines for all data points (solid) versus only adults 3m or larger as in Bianucci et al. (2023) (dashed) for *Trichechus* spp. Adult-based lines (dashed) vastly underestimate the intraspecific slope that is common across taxa.
