# Supplementary figures and images for: Downsizing a heavyweight: factors and methods that revise weight estimates of the giant fossil whale Perucetus colossus
Source: PeerJ. 2024 Feb 29;12:e16978. doi: 10.7717/peerj.16978 (PMC10909350; doi:10.7717/peerj.16978)

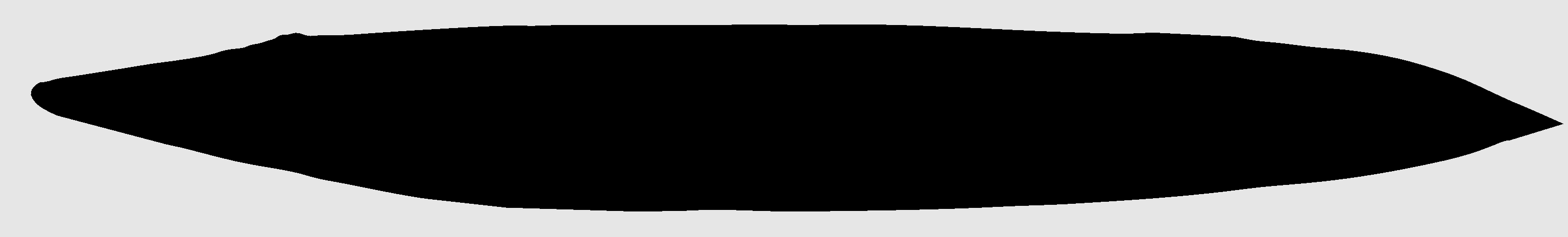

Supplement: Supplemental Information 1 [file peerj-12-16978-s001.zip › R/Balaenoptera_musculus/BodyL.png]

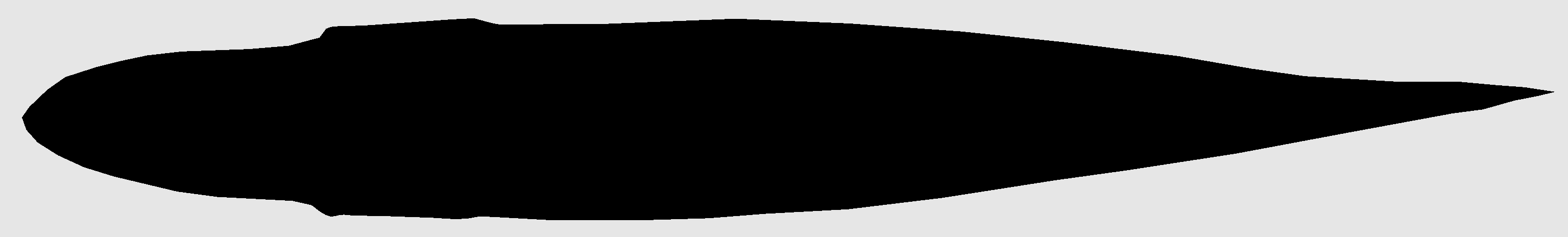

Supplement: Supplemental Information 1 [file peerj-12-16978-s001.zip › R/Balaenoptera_musculus/BodyV.png]

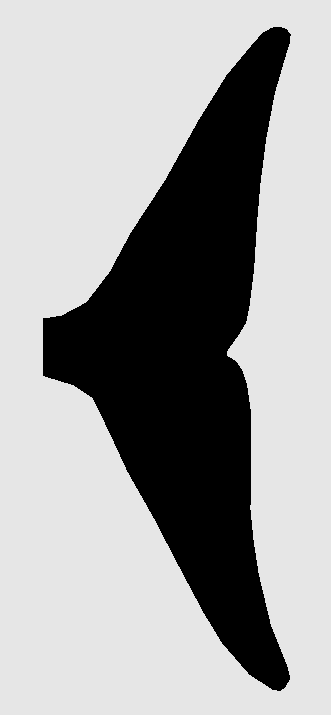

Supplement: Supplemental Information 1 [file peerj-12-16978-s001.zip › R/Balaenoptera_musculus/Cfin.png]

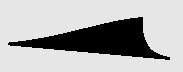

Supplement: Supplemental Information 1 [file peerj-12-16978-s001.zip › R/Balaenoptera_musculus/Dfin.png]

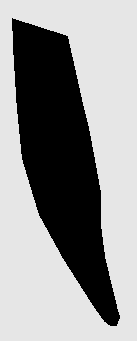

Supplement: Supplemental Information 1 [file peerj-12-16978-s001.zip › R/Balaenoptera_musculus/Ffin.png]

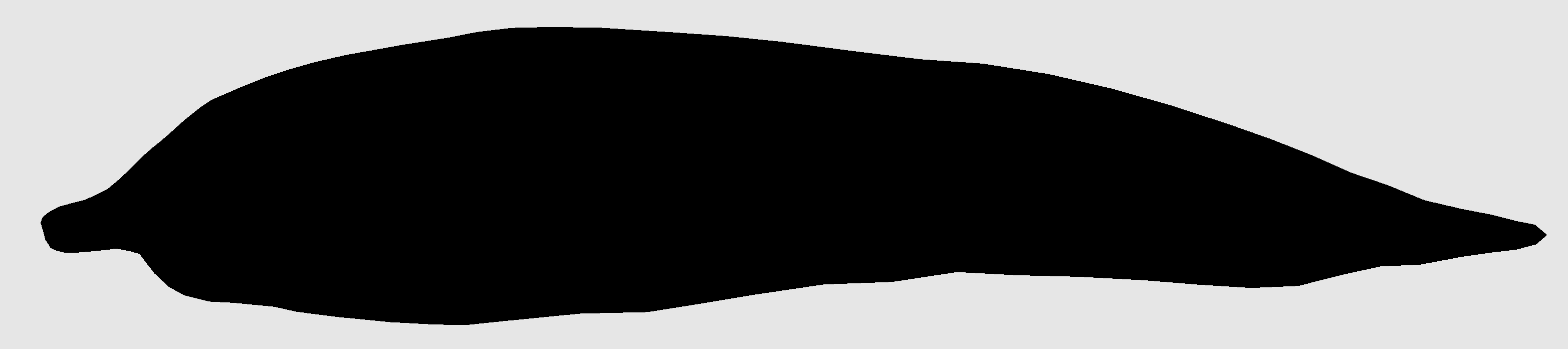

Supplement: Supplemental Information 1 [file peerj-12-16978-s001.zip › R/Perucetus/BodyL.png]

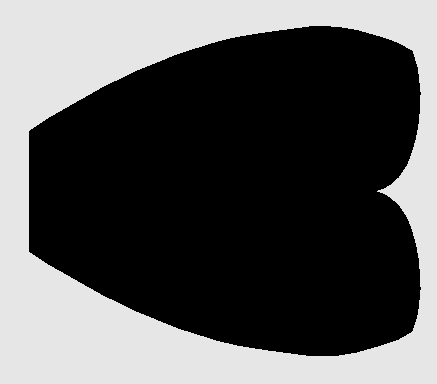

Supplement: Supplemental Information 1 [file peerj-12-16978-s001.zip › R/Perucetus/Cfin.png]

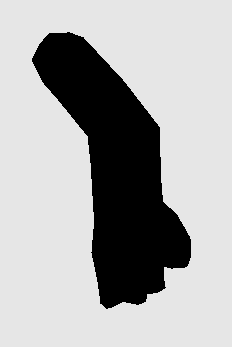

Supplement: Supplemental Information 1 [file peerj-12-16978-s001.zip › R/Perucetus/Ffin.png]

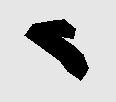

Supplement: Supplemental Information 1 [file peerj-12-16978-s001.zip › R/Perucetus/Hfin.png]
